# LncACTdb 2.0: an updated database of experimentally supported ceRNA interactions curated from low- and high-throughput experiments

**DOI:** 10.1093/nar/gky1144

**Published:** 2018-11-22

**Authors:** Peng Wang, Xin Li, Yue Gao, Qiuyan Guo, Yanxia Wang, Ying Fang, Xueyan Ma, Hui Zhi, Dianshuang Zhou, Weitao Shen, Weisha Liu, Lihua Wang, Yunpeng Zhang, Shangwei Ning, Xia Li

**Affiliations:** 1 College of Bioinformatics Science and Technology, Harbin Medical University, Harbin 150081, China; 2 Department of Neurology, The Second Affiliated Hospital, Harbin Medical University, Harbin 150081, China

## Abstract

We describe LncACTdb 2.0 (http://www.bio-bigdata.net/LncACTdb/), an updated and significantly expanded database which provides comprehensive information of competing endogenous RNAs (ceRNAs) in different species and diseases. We have updated LncACTdb 2.0 with more data and several new features, including (i) manually curating 2663 experimentally supported ceRNA interactions from >5000 published literatures; (ii) expanding the scope of the database up to 23 species and 213 diseases/phenotypes; (iii) curating more ceRNA types such as circular RNAs and pseudogenes; (iv) identifying and scoring candidate lncRNA-associated ceRNA interactions across 33 cancer types from TCGA data; (v) providing illustration of survival, network and cancer hallmark information for ceRNAs. Furthermore, several flexible online tools including LncACT-Get, LncACT-Function, LncACT-Survival, LncACT-Network and LncACTBrowser have been developed to perform customized analysis, functional analysis, survival analysis, network illustration and genomic visualization. LncACTdb 2.0 also provides newly designed, user-friendly web interfaces to search, browse and download all the data. The BLAST interface is convenient for users to query dataset by inputting custom sequences. The Hot points interface provides users the most studied items by others. LncACTdb 2.0 is a continually updated database and will serve as an important resource to explore ceRNAs in physiological and pathological processes.

## INTRODUCTION

Recently, accumulating evidence suggests that microRNAs (miRNAs) are themselves regulated by endogenous molecules carrying miRNA binding sites, such as long non-coding RNAs (lncRNAs), circular RNAs (circRNAs), pseudogenes, etc. Termed ‘miRNA sponges’, these competitive inhibitors bind miRNAs and competitively sequester them from their natural targets (1). The endogenous miRNA sponges and targets, also termed competing endogenous RNAs (ceRNAs), act to dynamically regulate the expression of each other in different physiological and pathological processes (2).

In recent years, many researchers have continued to focus on the influence of miRNA sponges or ceRNA interactions which uncover a novel mechanism underlying the regulation of oncogenes and tumor suppressors in various diseases including cancers (1,2). For example, the lncRNA H19 functions as a ceRNA to sponge miRNA let-7 family leading to an increase in expression of let-7 targets in breast cancer (3), ovarian cancer (4) and pancreatic cancer (5). A non-coding RNA PTENP1, which is a pseudogene of PTEN, has been found to act as a ceRNA and regulate PTEN levels by sponging common miRNAs in gastric cancer (6) and other cancers (7). In order to facilitate the study of miRNA sponges and ceRNA interactions, we reported the first version of the LncACTdb database (LncACTdb 1.0) which identifies active lncRNA–miRNA–mRNA interactions and can serve as a tool for dissecting the ceRNA regulation in various cancers and identifying novel cancer biomarkers (8). Although LncACTdb 1.0 has provided confidential resources for researchers, this database could provide more comprehensive information and be more user friendly. For example, LncACTdb 1.0 only focused on predicted ceRNA dataset of lncRNAs based on a limit number of 12 cancer types from The Cancer Genome Atlas (TCGA). In recent years, more and more ceRNA interactions across different RNA types have been identified by low- and high-throughput technologies. The incoming ceRNA datasets have enabled pan-cancer analysis to study the molecular alterations associated with different clinical outcomes (8,9). With the fast growing amount of both experimentally supported and predicted ceRNA data, there is a great need to update LncACTdb 1.0 with more resources and improved tools.

To date, several databases have been built to curate interactions between miRNAs and other molecules, such as starBase v2 (10), DIANA-LncBase v2 (11), miRSponge (12) and PceRBase (13). These databases have provided valuable resources for ceRNA studies. However, most of these databases predict interactions through a single target prediction method. The species in these databases are only restricted to human, mouse and plants. To our knowledge, only the miRSponge database stores experimentally supported datasets, with a limit number of 463 ceRNA associations in 11 species. Until now, no other specialized database has been devoted to collect, store and analyse experimentally supported ceRNA interactions, as well as comprehensive annotations.

To meet these needs, we have updated LncACTdb to version 2.0 (LncACTdb 2.0) with more data and several new features (Table 1). In comparison with LncACTdb 1.0, a number of 2663 experimentally supported ceRNA interactions across 23 species and >200 diseases/phenotypes have been manually curated and added to the database. Furthermore, the number of lncRNA associated-ceRNA interactions has been increased to 47 673. Expression profiles with clinical information of more than ten thousands cancer patients from TCGA have been integrated into LncACTdb 2.0. LncACTdb 2.0 also provides illustration of survival, network and cancer hallmark information for ceRNAs. In addition to the expansion of the core data sets, LncACTdb 2.0 provides newly designed, user-friendly web interfaces to query, analyse and download all the data. In particular, several flexible online tools have been developed to facilitate data extraction, analysis and visualization. Collectively, we expect this updated database could facilitate the identification of disease associated ceRNAs and benefit the investigation of their roles in physiological and pathological processes. All the information in LncACTdb 2.0 is freely available at http://www.bio-bigdata.net/LncACTdb.

**Table 1.** Content and statistics of LncACTdb 2.0

Datasets and features		LncACTdb 1.0	LncACTdb 2.0	Fold increase
Experimentally supported dataset	CeRNA interactions	No	2663	New
	LncRNAs	No	312	New
	MiRNAs	No	479	New
	CircRNAs	No	59	New
	MRNAs	No	131	New
	Pseudogenes	No	16	New
	Viral RNAs	No	9	New
	Artificially engineered RNAs	No	105	New
	Species	No	23	New
	Diseases/Phenotypes	No	213	New
Predicted dataset	CeRNA interactions	5119	47 673	9.31
	MiRNA–lncRNA interactions	1890	3181	1.68
	MiRNA–mRNA interactions	1229	7006	5.70
	LncRNAs	335	1191	3.56
	MiRNAs	212	502	2.37
	MRNAs	1312	2792	2.13
	Cancer types	12	33	2.75
Annotations and analyzing tools	Pan-cancer information	No	Yes	New
	MiRNA binding information	No	Yes	New
	Experimental methods	No	Yes	New
	Network illustration	No	Yes	New
	Functional annotations	Yes	Yes	New
	Cancer Hallmarks	No	Yes	New
	Survival analysis	No	Yes	New
	BLAST tool	No	Yes	New
	Online data-mining	Yes	Yes	New
	Genome browser	Yes	Yes	New

## IMPROVED CONTENTS AND NEW FEATURES

### Newly added entries of experimentally supported ceRNAs

High-confidence ceRNA interactions were manually curated from literatures and integrated into LncACTdb 2.0 database. In this update, we retrieved published literatures from PubMed by employing key words related with ceRNAs and found >5000 relevant articles (before October 2018, Supplementary Methods). We classified these articles by years and found that the number of records were significantly increased in recent years, especially from 2017 to 2018 (Supplementary Figure S1). The rapid growing number of ceRNA related literatures indicates the urgent need to collect corresponding dataset and update the LncACTdb database. In this work, we manually collected experimentally supported ceRNAs through several steps as previously described (14). The experimentally supported ceRNA associations were manually curated from these published articles by at least two researchers. Only datasets supported by high confidence experiments such as PCR, western blot, or luciferase reporter assay, and other reliable methods were considered and further curated. The exact description of the experimental types, curation process and criteria used in our pipeline were shown in Supplementary Methods. Currently, LncACTdb 2.0 documents a total of 2663 experimentally supported ceRNA interactions, including 312 lncRNAs, 131 coding mRNAs, 59 circRNAs and 16 pseudogenes. The scope of LncACTdb 2.0 is expanded to 23 species and 213 diseases/phenotypes.

### Expanded entries of lncRNA-associated ceRNAs in pan-cancers

**Figure 1.**
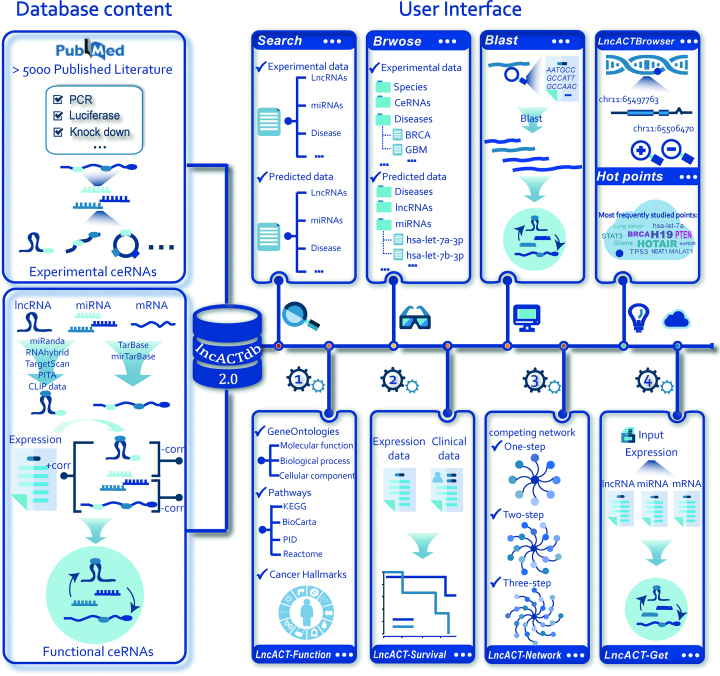
Content and interface of LncACTdb 2.0. The left panel is the database content which including ceRNA information identified from low- and high-throughput experiments. The right panel is the user interface of LncACTdb 2.0. In this panel, the Search, Browse, Blast and LncACTBrowser models provide flexible ways to access the dataset. The online tools including LncACT-Function, LncACT-Survival, LncACT-Network and LncACT-Get have been developed to perform customized analysis and data visualization.

In recent years, lncRNAs have been widely reported to be involved in ceRNA regulations in order to communicate with other RNA transcripts in a wide range of diseases (15–17). To provide a comprehensive resource of lncRNA-associated ceRNA regulations across different cancers, we used an integrative pipeline (8) to identify candidate lncRNA-associating ceRNAs from TCGA (Figure 1, Supplementary Methods). The miRNA–lncRNA interactions were predicted using four miRNA target prediction method with strict thresholds. Further, 41 AGO-CLIP-seq datasets downloaded from starBase v2 (10) were integrated into the pipeline to identify experimentally supported miRNA-binding sites on lncRNA sequences. Genomic coordinates of CLIP-seq peaks and predicted miRNA-binding sites were compared by BEDTools (18) using length of overlap >1 as threshold. The miRNA-mRNA regulations which were validated by strong experimental methods such as luciferase reporter assay, PCR and Western blot were derived from TarBase (v8) (19) and mirTarBase (v2018) (20). If a lncRNA and mRNA interacting with the same miRNA, this lncRNA-miRNA-mRNA competing triplet was termed as a candidate ceRNA interaction. A functional ceRNA was defined for a certain cancer type if it met all of the following criteria: *corr*(*lncRNA, miRNA*) < 0, *corr*(*mRNA, miRN*A) < 0 and *corr*(*lncRNA, mRNA*) > 0, where *corr*(*a, b*) representing the Pearson correlation coefficient of gene *a* and *b* based on their expression values, respectively. In LncACTdb 2.0, the cancer types have been expanded from 12 to 33. Ultimately, 47 673 functional ceRNAs with competing activity score across pan-cancers were identified in LncACTdb 2.0. To facilitate the study of ceRNAs, LncACTdb 2.0 provides detail information of miRNA binding sites for miRNA–lncRNA interactions and experimentally validated methods for miRNA–mRNA interactions.

### Expanded contexts of networks, functions, hallmarks and prognosis

For each lncRNA-associated ceRNA entry, LncACTdb 2.0 constructs a network consisting of this ceRNA and its associated competing neighbors and further provides a graphic illustration. A ‘guilt-by-association’ strategy has been used in LncACTdb 2.0 to perform functional annotation of ceRNAs (Supplementary Methods). Currently, more functional contexts have been added to LncACTdb 2.0 for providing a comprehensive annotation background. For pathway annotation, a total number of 1329 pathways including KEGG (21), BioCarta (https://cgap.nci.nih.gov/Pathways), Reactome (22), PID (23), STKE (http://stke.sciencemag.org/) and SIG (http://www.signaling-gateway.org/) were downloaded from MSigDB (24). For pathways in each database, we collected Entrez IDs as functional gene lists. For Gene Ontology annotation (25), a total number of 5917 gene sets representing functional terms were collected. The Entrez IDs in each GO terms were used as functional gene lists. Gene sets of cancer hallmark processes, which have been determined to promote tumor growth and metastasis (24,26), have been downloaded from MSigDB. For survival analysis, clinical follow-up information of 10 141 patients from TCGA were collected. A risk score model was constructed according to the linear combination of ceRNAs expression values weighted by the Cox regression coefficient (8). Further, the median or mean risk score was used as a cut-off to divide patients into two groups with different survival risk. The Kaplan-Meier survival analysis was performed for the two groups of patients, and statistical significance was assessed using log-rank test (*P* < 0.05).

### Newly developed tools for data discovery and analysis

With the fast growing number of expression profiles resulting from high-throughput technologies, there are urgent need to analyse these large amount datasets for dissecting disease pathology and discovering cancer biomarkers. In LncACTdb 2.0, we have updated the LncACT-Get tool for users to identify novel ceRNA interactions according to customized input. Users can upload the expression profiles of a certain disease or phenotype, and LncACT-Get will implement an integrated pipeline to identify functional ceRNA interactions with corresponding activity scores and *P* values. To study the downstream biological processes affected by lncRNAs, the LncACT-Function tool performs functional analysis of user inputted lncRNAs based on ‘guilt-by-association’ strategy. LncACT-Function tool collected thousands of pathways and biological terms as functional background. To discover novel prognostic ceRNA biomarkers, we developed the LncACT-Survival tool, which performs online survival analysis for a certain ceRNA interaction across 33 cancer types of TCGA. In addition, LncACT-Survival tool also provides survival analysis for a single lncRNA, miRNA or mRNA. To facilitate visualization of ceRNA networks, the LncACT-Network tool has been newly developed. For a customized lncRNA or mRNA, LncACT-Network tool will provide a global view of all possible ceRNA interactions and more detail information on cross-talk between different ceRNAs.

### More flexible ways to access the dataset

The LncACTdb 1.0 database has provided user-friendly interfaces for database queries, such as Search and Browse pages. Because of the fast growing number of data entries and newly identified RNA sequences, LncACTdb 2.0 provides more flexible ways for data discovery and access: (i) A quick search engine has been developed which allows users to search both the experimentally supported and predicted dataset. The inputted key words can be any of lncRNAs, miRNAs, mRNAs, circRNAs, pseudogenes, diseases, cell lines, primary sites and etc. The search engine supports fuzzy searching, which will list all potential results matching the key words. (ii) A new data access tool named BLAST has been developed to implement a customized sequencing search. Users can input new RNA sequences in order to identify related ceRNAs. (iii) The Hot points interface reviews the visited records of LncACTdb 2.0 and provides users the most studied items by other researchers. (iv) LncACTBrowser is a web-based genome browser that dynamically displays different tracks for ceRNAs. It provides comprehensive tracks including reference sequence, transcripts, miRNA-binding sites (predicted by miRanda, TargetScan, PITA and RNAhybrid method) and CLIP-seq peaks (41 datasets). (v) The customized results can be flexibly downloaded on touch of the ‘Copy’, ‘Excel’ and ‘CSV’ buttons through all querying steps. In addition, all associated datasets can be freely downloaded in Download page.

## DATABASE CONSTRUCTION AND IMPROVED USER INTERFACE

All data in LncACTdb 2.0 were documented and managed in MySQL database (v 5.5). The web server was updated by using Java Server Pages within Tomcat container (v6). The LncACTdb 2.0 database is freely available at http://www.bio-bigdata.net/LncACTdb/. In addition, for the convenience of users who have used LncACTdb 1.0, the old version is still in service. Users can enter it by clicking on the gateway in LncACTdb 2.0 homepage or go directly to http://www.bio-bigdata.net/LncACTdb1.0.

**Figure 2.**
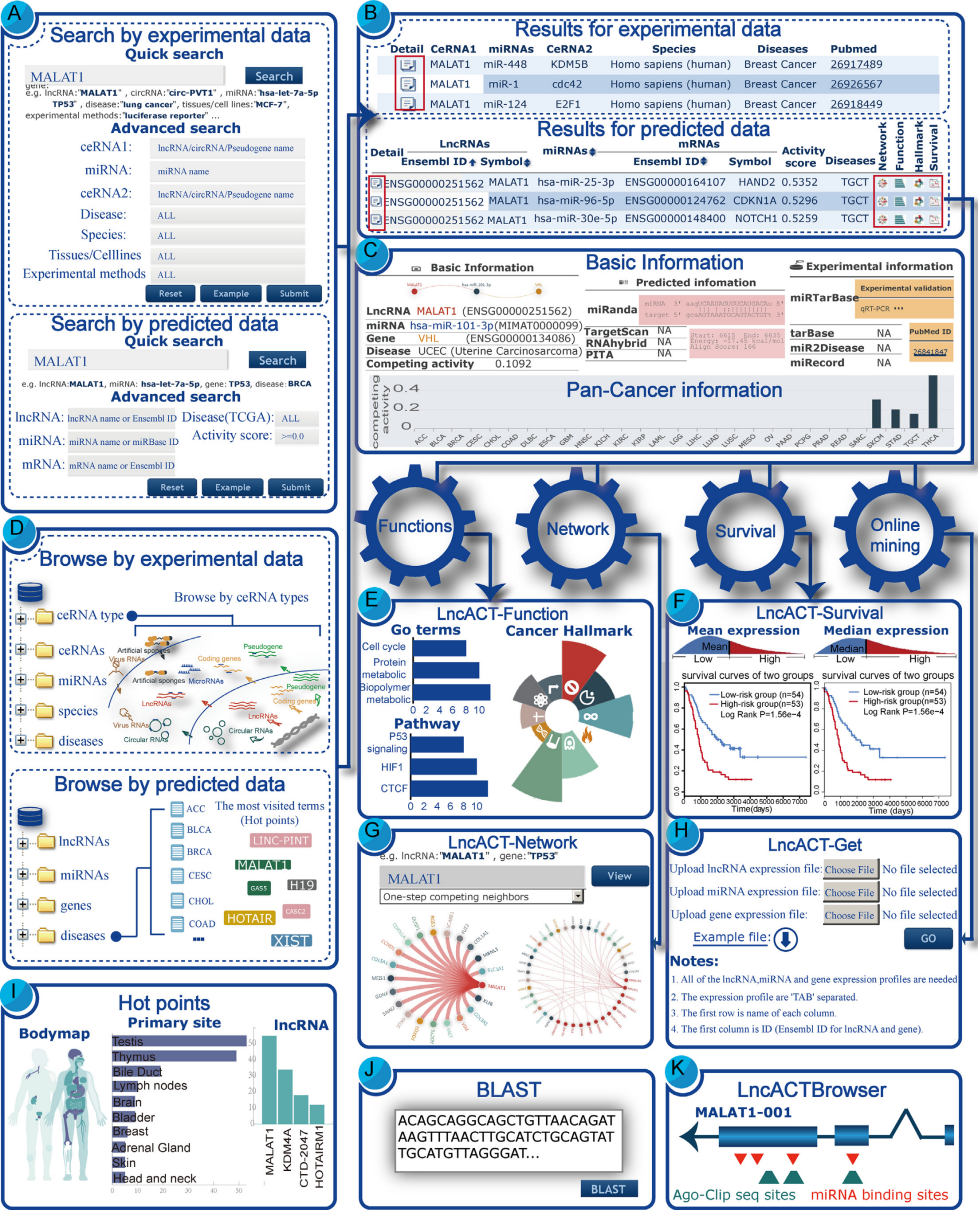
Case study and workflow of using LncACTdb 2.0. (**A**) The interface of the search module with an example of MALAT1. (**B**) The search results of MALAT1, including both predicted and experimentally supported dataset. (**C**) Search result page with detail information. (**D**) The browse interface of LncACTdb 2.0. (**E**) Functional analysis of MALAT1 based on context of GO terms, pathways and cancer hallmarks. (**F**) Survival analysis and Kaplan–Meier survival curves for MALAT1-associating ceRNAs. (**G**) A global view of all possible related ceRNA interactions for MALAT1. (**H**) The LncACT-Get tool implements an integrated pipeline to identify functional ceRNA interactions according to customized data. (**I**) The Hot points page provides a human body map and the most studied items by other researchers. (**J**) The BLAST interface implements a customized sequencing search to identify related ceRNAs. (**K**) The LncACTBrowser which provides comprehensive genomic information of MALAT1, including reference sequence, transcripts, miRNA-binding sites and CLIP-seq peaks.

LncACTdb 2.0 provides a user-friendly web interface that enables users to search, browse, analyse and download data in a few easy steps (Figure 2). As an example of lncRNA MALAT1 inputted in the search interface (Figure 2A), all possible ceRNA interactions will be displayed in the results page (Figure 2B). To filter out interesting ceRNA interactions, users can reorder the result table by clicking on the header of different columns. The first column will lead users to the detail information page for each ceRNA interaction. LncACTdb 2.0 provides panels of comprehensive information including basic information, pan-cancer information, predicted and experimental information for the MALAT1-associating ceRNA interaction (Figure 2C). To further analyse the dataset, several online tools have been developed and can be easily accessed on the navigation bar of every page (Figure 2E–H). The LncACT-Function tool performs functional analysis of MALAT1 based on GO terms, pathways and cancer hallmarks (Figure 2E). The LncACT-Survival tool performs survival analysis and provides Kaplan–Meier survival curves for each competing partners and the whole ceRNA interaction (Figure 2F). The LncACT-Network tool provides a global view of all possible related ceRNA interactions (Figure 2G). Users can reset the scale of the network by adjusting different steps of neighbors. According to customized expression profiles, the LncACT-Get tool implements an integrated pipeline to identify functional ceRNA interactions with corresponding activity scores and *P* values (Figure 2H). In addition, LncACTdb 2.0 provides more flexible ways to access the dataset. A Browse page was designed for general perusal of the database based on different classifications (Figure 2D). The Hot points page provides a human body map illustration and the most studied items by other researchers (Figure 2I). The BLAST page implements a customized sequencing search. Users can input new RNA sequences in order to identify related ceRNAs (Figure 2J). The LncACTBrowser is a web-based genome browser which provides comprehensive tracks including reference sequence, transcripts, miRNA-binding sites and CLIP-seq peaks information (Figure 2K).

## CONCLUSIONS AND FUTURE DEVELOPMENT

In the first version of LncACTdb database (LncACTdb 1.0), only a limited number of predicted ceRNA interactions have been identified in human. With the development of high-throughput sequencing technology and experimental validation method, the number of ceRNAs were significantly increased in recent years, especially from 2017 to 2018 (Supplementary Figure S1). The fast growing number of related literatures indicates the urgent need to collect corresponding dataset and update the first version of LncACTdb database. Currently, the datasets and functions of LncACTdb 2.0 have been significantly improved. The scope of LncACTdb 2.0 is expanded to 23 species and 213 associating diseases/phenotypes. To provide a comprehensive source of ceRNA regulations with disease information, we developed an integrative pipeline to identify candidate lncRNA-associating ceRNA regulations (Figure 1). A total number of 47 673 predicted ceRNA interactions across 33 types of cancers of TCGA have been provided by LncACTdb 2.0. Based on clinical follow-up information of 10 141 TCGA cancer patients, the prognostic information including Cox regression coefficients and Kaplan-Meier survival curves have also been provided. To improve the functions of data access and analysis, more function contexts such as biological pathways and cancer hallmarks have been integrated into LncACTdb 2.0. We expect that the number of ceRNA datasets identified from high-confidence experiments or high-throughput analysis will continue to increase rapidly in the future releases of LncACTdb database. We will continually maintain and update the LncACTdb database with more data sets and functional interfaces, which will improve our understanding of the coding and non-coding RNAs in complex diseases.

## Supplementary Material

Supplementary Data
