# Supplementary material for: LncACTdb 2.0: an updated database of experimentally supported ceRNA interactions curated from low- and high-throughput experiments
Source: Nucleic Acids Res. 2018 Nov 22;47(Database issue):D121–7. doi: 10.1093/nar/gky1144 (PMC6324071; doi:10.1093/nar/gky1144)
Supplement: Supplementary Data [file gky1144_supplemental_files.pdf]

Supplementary Methods

Identification of functional lncRNA-associated ceRNAs in pan-cancers

To provide a comprehensive resource of lncRNA-associated ceRNA regulations across different cancers, we used an integrative pipeline to identify candidate lncRNA-associating ceRNAs across 33 cancer types of TCGA. Comprehensive genome annotations of 19,817 protein-coding mRNAs and 27,720 lncRNAs from GENCODE (v26, GRCH38) were collected. A number of 2,588 mature miRNA sequences were downloaded from miRBase database (v21). The miRNA-lncRNA interactions were predicted using four miRNA target prediction method: miRanda (v2010) (1), TargetScan (v.6.0) (2), PITA (v2007) (3) and RNAhybrid (v.2.1.1) (4) with strict thresholds (miRanda: score>160 and energy<-20; TargetScan: context score<-0.4; PITA: ddg<-10; RNAhybrid: mfe<-25 and P<0.01). Further, 41 AGO-CLIP-seq datasets downloaded from starBase v2.0 (5) were integrated into the pipeline to identify experimentally supported miRNA-binding sites on lncRNA sequences. We used the criterion that peaks derived from the CLIP experiments suggest direct binding sites of miRNAs (6). We compared the genomic coordinates of CLIP-seq peaks and predicted miRNA-binding sites by using BEDTools software (7). The threshold was set as length of overlap >1. A number of 185,662 miRNA-lncRNA interactions were retained for further analysis. The miRNA-mRNA regulations were derived from TarBase (v8) (8) and mirTarBase (v2018) (9), which store manually curated collections of experimentally supported miRNA targets. A number of 10,089 miRNA-mRNA interactions only validated by strong experimental methods such as luciferase reporter assay, PCR and Western blot were collected into LncACTdb 2.0. If a lncRNA and mRNA interacting with the same miRNA, this lncRNA-miRNA-mRNA competing triplet was termed as a candidate ceRNA interaction. A functional ceRNA was defined for a certain cancer type if it met all of the following criteria: corr(*lncRNA, miRNA*) < 0, corr(*mRNA, miRNA*) < 0 and corr(*lncRNA, mRNA*) > 0, where corr (*a*, *b*) representing the Pearson correlation coefficient of gene *a* and *b* based on their expression values, respectively. The significance threshold for correlation analysis was set as FDR<0.05. Because the miRNA expression datasets of GBM were not provided by TCGA, we only calculated the corr(*lncRNA, mRNA*) value for each candidate ceRNA interaction of GBM with strict threshold: FDR<0.01 and corr(*lncRNA,*

mRNA)>0.5. Ultimately, 47,673 functional ceRNAs comprising 1,191 lncRNAs, 502 miRNAs, and 2,792 mRNAs were identified in LncACTdb 2.0.

The competing activity score

Expression correlation is a statistically useful method for discerning the association between direct and indirect interacting RNAs and is robust to different normalization methods between mRNA-seq and miRNA-seq data (10). To characterize the strength of competition in ceRNA interactions, the competing activity score was defined as the average value of corr *(lncRNA, miRNA*), corr (*mRNA, miRNA*) and corr (*lncRNA, mRNA*). For GBM dataset, we used the corr(*lncRNA, mRNA*) values as the activity scores (FDR<0.01). A higher competing activity score indicating greater competition between the lncRNA and mRNA for miRNA binding. For each score, the maximum Pearson correlation P-value was considered as the overall P value of a ceRNA interaction.

Identification of functional ceRNAs from user uploaded datasets

We have updated the LncACT-Get tool for users to identify novel ceRNA interactions according to customized input. Users can upload the expression profiles of a certain disease or phenotype, and LncACT-Get will implement the above pipeline to identify functional ceRNA interactions with corresponding activity scores. To test the significance of activity scores, LncACTdb 2.0 performs benchmark analysis to calculate empirical P values. In this step, expression profiles of lncRNAs, miRNAs and mRNAs are randomly shuffled by rows and columns to generate a random competing activity score. This is compared to random scores 10,000 times, with the P value representing the percentage of times that a random score is higher than the competing activity score.

Manual curation of high-confidence experimentally verified ceRNAs

To expand the scope of LncACTdb 2.0, we performed manually curation of experimentally verified ceRNA interactions from published literatures. In this update, we retrieved published literatures from PubMed database by employing key words including "miRNA sponges", "ceRNA", "miRNA decoy", "competing RNA", "antagomir", "miRNA mediated", "miRNA target lncRNA", "miRNA target nc-RNA", "miRNA target circular RNA", "miRNA target pseudogene"

and found more than 5,000 relevant articles (before Oct 2018). The full list of key words and related articles can be downloaded at http://www.bio-bigdata.net/LncACTdb/download.jsp. All selected articles were reviewed by several researchers. In this step, the searchers will confirm that if the regulation relationship between each competing RNA members were verified by one of the high-confidence experimental methods, including RT-PCR, qRT-PCR, western blot, Northern blot, luciferase reporter assay, RNAi, in vitro knockdown, RNA immunoprecipitation and cell assay. Our researchers filter the ceRNA associations following the criteria: (i) if the RNAs within a ceRNA interaction act to dynamically regulate the expression of each other, (ii) if the RNAs within a ceRNA interaction share the same miRNA binding site and (iii) ceRNA entries that were simultaneously confirmed by at least two researchers were retained. We retrieved more ceRNA types such as lncRNAs, coding-mRNAs, pseudogenes, lncRNAs, circular RNAs, intruded viral RNAs and technically engineered RNAs. Detail information including members of ceRNAs, species, tissues/cell lines, disease/phenotype, experimental methods, PubMed ID, paper title and a functional description from the original studies. Currently, LncACTdb 2.0 documents a total of 2,663 high-confidence experimentally verified ceRNAs interactions. The scope of LncACTdb 2.0 is expanded to 23 species and 213 associating diseases/phenotypes.

Functional analysis of lncRNAs based on ceRNA theory

LncACTdb 2.0 provides the LncACT-Function tool to perform functional analysis of lncRNAs based on 'guilt-by-association' strategy. For a lncRNA, its competing coding-mRNA partners were used to performed function enrichment analysis. LncACT-Function tool curated thousands of pathways and biological terms as functional background. For pathway annotation, a total number of 1,329 pathways including KEGG (11), BioCarta (https://cgap.nci.nih.gov/Pathways), Reactome (12), PID (13) , STKE (http://stke.sciencemag.org/) and SIG (http://www.signaling-gateway.org/) were downloaded from MSigDB (14). For pathways in each database, we collected Entrez IDs as functional gene lists. For Gene Ontology annotation, a total number of 5,917 gene sets representing functional terms were collected. Gene sets of cancer hallmark processes, which have been determined to promote tumor growth and metastasis (15), have been downloaded from MSigDB (14)

which performs manual curation based on several steps: (i) Identify groups of similar gene sets using consensus clustering. (ii) Filter clusters and identify biological themes. (iii) Identify gene expression datasets for refinement. (iv) Define raw hallmark sets. (v) Refining raw hallmark sets. (vi) Independent validation and final hallmark set. Hypergeometric test was used to calculate the enrichment significance based on different functional gene sets. If the genome context had a total of *N* genes, of which *S* were involved in the function set under investigation, and the set of interesting target genes for analysis had a total of *M* genes, of which *x* were involved in the same function gene set, then the P value can be calculated as:

$$P = 1 - \sum_{t=0}^{x} \frac{\binom{S}{t}\binom{N-S}{M-t}}{\binom{N}{M}}$$

Significantly enriched functions was defined at P<0.05 level and further illustrated as bar graph based on –log 10 transformed P values.

Survival analysis of ceRNAs

The LncACT-Survival tool performs survival analysis and provides Kaplan-Meier survival curves for each competing partners and the whole ceRNA interaction. For survival analysis, clinical follow-up information of 10,141 patients from TCGA were collected. A univariate Cox regression analysis was carried out to evaluate the association between survival and expression level of each lncRNA-miRNA-mRNA member in a ceRNA interaction. A risk score formula was developed to evaluate the association between survival and expression in a certain cancer, which takes into account both the strength and positive/negative association between each competing RNA and probability of survival. The integrated risk score for each patient was calculated based on the linear combination of ceRNAs expression values weighted by the Cox regression coefficients:

$$Risk\ socre = \sum_{i=1}^{n} r_i Exp(i)$$

where r_i is the Cox regression coefficient of a lncRNA, miRNA or mRNA in a ceRNA interaction, *n* is the number of competing RNAs, and $Exp(i)$ is the expression value of RNA i in corresponding patient. The median and mean risk score was used as cut-off to classify

patients into high and low-risk groups.

Network construction of ceRNAs

The LncACT-Network tool provides a global view of all possible related ceRNAs relationships. For each lncRNA-associated ceRNA entry, LncACTdb 2.0 constructs a network consisting of this ceRNA and its associated competing neighbors and further provides a graphic illustration. Users can reset the scale of the network by adjusting different steps of neighbours. In the one-step-neighbours scale, the top 20 competing mRNA partners (ordered by activity score) of the lncRNA were illustrated. In the two-step-neighbours and three-step-neighbours scale, this network will expand to another 20 and 40 competing lncRNAs and mRNAs.

The BLAST method

The BLAST tool is convenient for users to query dataset by inputting custom sequences. LncACTdb 2.0 implements the Basic Local Alignment Search Tool (BLAST, https://blast.ncbi.nlm.nih.gov/Blast.cgi) to compare inputting sequence to our database and calculates the statistical significance of matches. The parameters were set as -perc_identity 80 -outfmt "7 qacc sacc evalue length pident". Both predicted and experimentally validated ceRNA transcripts which had high similarity (>80% identity) with inputting sequence will be listed in a new page.

REFERENCES

1. Betel, D., Wilson, M., Gabow, A., Marks, D.S. and Sander, C. (2008) The microRNA.org resource: targets and expression. *Nucleic Acids Res*, **36**, D149-153.
2. Friedman, R.C., Farh, K.K., Burge, C.B. and Bartel, D.P. (2009) Most mammalian mRNAs are conserved targets of microRNAs. *Genome Res*, **19**, 92-105.
3. Kertesz, M., Iovino, N., Unnerstall, U., Gaul, U. and Segal, E. (2007) The role of site accessibility in microRNA target recognition. *Nat Genet*, **39**, 1278-1284.
4. Rehmsmeier, M., Steffen, P., Hochsmann, M. and Giegerich, R. (2004) Fast and effective prediction of microRNA/target duplexes. *Rna*, **10**, 1507-1517.
5. Li, J.H., Liu, S., Zhou, H., Qu, L.H. and Yang, J.H. (2014) starBase v2.0: decoding miRNA-ceRNA, miRNA-ncRNA and protein-RNA interaction networks from large-scale CLIP-Seq data. *Nucleic acids research*, **42**, D92-97.
6. Paraskevopoulou, M.D., Vlachos, I.S., Karagkouni, D., Georgakilas, G., Kanellos, I., Vergoulis, T., Zagganas, K., Tsanakas, P., Floros, E., Dalamagas, T. *et al.* (2016) DIANA-LncBase v2: indexing

microRNA targets on non-coding transcripts. *Nucleic acids research*, **44**, D231-238.
7. Quinlan, A.R. and Hall, I.M. (2010) BEDTools: a flexible suite of utilities for comparing genomic features. *Bioinformatics*, **26**, 841-842.
8. Karagkouni, D., Paraskevopoulou, M.D., Chatzopoulos, S., Vlachos, I.S., Tastsoglou, S., Kanellos, I., Papadimitriou, D., Kavakiotis, I., Maniou, S., Skoufos, G. *et al.* (2018) DIANA-TarBase v8: a decade-long collection of experimentally supported miRNA-gene interactions. *Nucleic acids research*, **46**, D239-D245.
9. Chou, C.H., Shrestha, S., Yang, C.D., Chang, N.W., Lin, Y.L., Liao, K.W., Huang, W.C., Sun, T.H., Tu, S.J., Lee, W.H. *et al.* (2018) miRTarBase update 2018: a resource for experimentally validated microRNA-target interactions. *Nucleic acids research*, **46**, D296-D302.
10. Cho, S., Jang, I., Jun, Y., Yoon, S., Ko, M., Kwon, Y., Choi, I., Chang, H., Ryu, D., Lee, B. *et al.* (2013) MiRGator v3.0: a microRNA portal for deep sequencing, expression profiling and mRNA targeting. *Nucleic Acids Res*, **41**, D252-257.
11. Kanehisa, M., Goto, S., Sato, Y., Furumichi, M. and Tanabe, M. (2012) KEGG for integration and interpretation of large-scale molecular data sets. *Nucleic acids research*, **40**, D109-114.
12. Croft, D., Mundo, A.F., Haw, R., Milacic, M., Weiser, J., Wu, G., Caudy, M., Garapati, P., Gillespie, M., Kamdar, M.R. *et al.* (2014) The Reactome pathway knowledgebase. *Nucleic acids research*, **42**, D472-477.
13. Schaefer, C.F., Anthony, K., Krupa, S., Buchoff, J., Day, M., Hannay, T. and Buetow, K.H. (2009) PID: the Pathway Interaction Database. *Nucleic acids research*, **37**, D674-679.
14. Liberzon, A., Birger, C., Thorvaldsdottir, H., Ghandi, M., Mesirov, J.P. and Tamayo, P. (2015) The Molecular Signatures Database (MSigDB) hallmark gene set collection. *Cell systems*, **1**, 417-425.
15. Hanahan, D. and Weinberg, R.A. (2011) Hallmarks of cancer: the next generation. *Cell*, **144**, 646-674.

Supplementary Figures

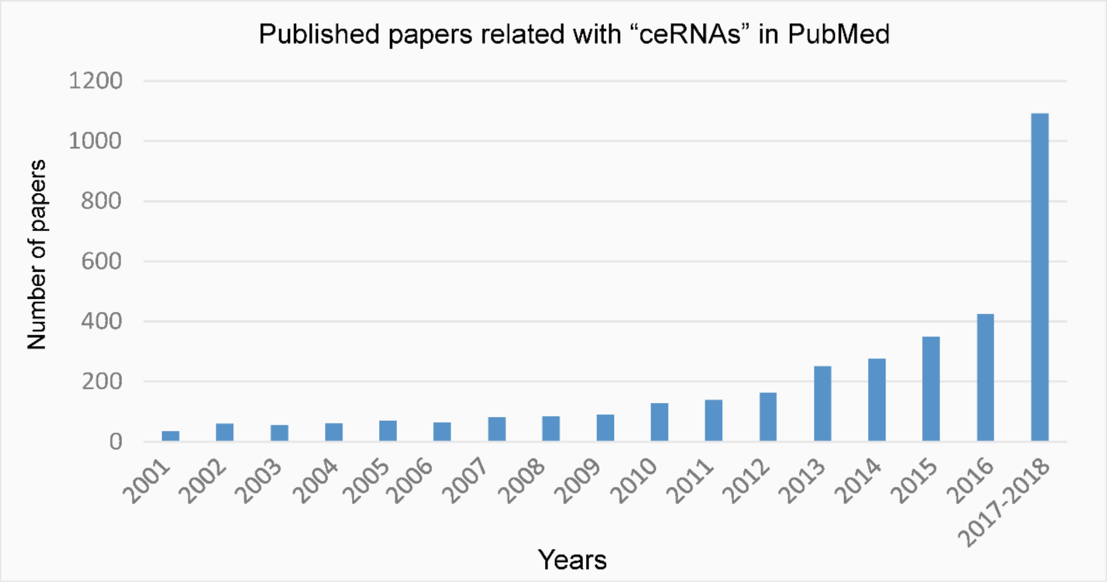

Figure S1. Published papers related with ceRNAs in recent years.
